# On the Action of Subnitrate of Bismuth

**Published:** 1853-07

**Authors:** Filippo Lussanna


From the Gazzetta Medica Toscana, p 44.—Dublin Quarterly Jour., Feb. 1853

*On the action of the Subnitrate of Bismuth.* By Dr. Filippo Lussanna.

The subnitrate of bismuth, a medicine already well known, has been lately used with new indications and pharmacological applications. Notwithstanding the fear which previously existed of its local caustic action. Monneret has recently recommended it in large doses as a topical (internal) anodyne in various gastro-intestinal affections, gastralgia, diarrhœa, dyspepsia and cholerine. He, following the fashion of many of his countrymen, ignored almost every general action of this preparation, and declared that its therapeutic efficacy is purely local, and that, far from being irritating, it is absorbent and calming. Monneret has rendered much service to science, by destroying, so far as this remedy is concerned, the Orfillian scare-crows which still not a little impede the rational interpretation of pharmacological facts. But, at the same time, I do not believe that all his deductions are correct and true, when he entirely denies the general effects of this metal. Following his directions, I have administered the subnitrate of bismuth in the large doses given by him. The patients so treated were affected with tubercular diarrhœa, the consequence of chronic enteritis, gastralgia of long standing and mesenteric disease. From the study of the effects of this remedy, I have been able to deduce the following inferences,

1. It is true that it does not give rise to intestinal irritation.

2. I have not seen it arrest tubucular or mesenteric diarrhœa.

3. The fæces always acquired a blackish-yellow color, owing to the unassimilated portions of the medicine being converted into sulphuret of bismuth by contact with the sulphuretted hydrogen of the fæcal masses.

4. The excrement, although still presenting the character of diarrhœa, was always rendered less liquid in consequence of admixture with the powder.

5. The subnitrate of bismuth is in part assimilable, and some portion of the large quantity swallowed is really dissolved and absorbed. This takes place with many other medicines, of which only a portion can be dissolved or absorbed, while the remainder passes unacted on, when the remedy has been given in large doses, through the intestine. Such is the case with calomel, iron and kermes, among metallic preparations.

6. The assimilation of the subnitrate of bismuth is due to the acid action of the fluids of the stomach, which render it soluble. Any portion that reaches the intestine, whether undissolved or dissolved, cannot be digested or absorbed, because the alkaline chlorides of the intestinal tube have no solvent effect upon it, and precipitate it if dissolved. In this second point of view, Monneret would be quite right in denying the effects of this salt on the economy in general, and in denying its absorption. The subnitrate of bismuth is soluble in acidulous matters, and, consequently, can be digested in the stomach. Thus we can understand how Trousseau and Pidoux, who were also ignorant of the general action of the salt, should, notwithstanding, caution us against administering it when gastric affections are attended with eructations,—in which, say they, "the medicine almost always fails," since, by rendering it more soluble and assimilable, the presence of medicine developes its dynamic action.

7. The non-appearance of the subnitrate of bismuth in the urine does not exclude the possibility of its having been introduced into the circulation. In fact, the subnitrate, having entered the current of blood, is, by the action of the alkaline chlorides of the serum, restored to the state of an insoluble subsalt, and thus becomes incapable of passing off by the uriniferous emunctory.

8. The effects of bismuth thus introduced into the economy are colliquative and scorbutic. The patient acquires a leaden aspect, the eyes become sunken, and present a livid subpalbebral circle; the breath is rendered offensive, the gums swell, grow livid, and discharge a sanious blood; hemorrhage is easily excited, and sometimes profuse passive hemorrhages arise. I had once under my care a case of a dangerous and repeated epistaxis produced by it in mesenteric tuberculosis. Sometimes blood is seen in the expectoration; finally, the alvine dejections are occasionally melenic. Everything announces a solvent action on the globulin, similar to that produced, according to the experiments of Dumas, by the chlorides of potassium, sodium, and ammonium, which dissolve blood corpuscles. It might be classed among Miahle's fluidifying agents.

9. Therefore, when we wish to prevent the absorption, or the true medical action of the subnitrate of bismuth, to localize its action, and produce none but its mechanical absorbent effects, the rational mode will be to precede or accompany its administration by the exhibition of an antacid, as, for example, calcined magnesia, so as to neutralize and fix the solvent acid gastric fluids. And this, of course, will also be the chemical antidote of the preparations of bismuth, with the view of preventing their assimilation, while the preparations of iron and stimulants will be useful in counteracting their general or dynamic effects of producing scurvy,—fluidifying and dissolving the hematin.

